# Exploring Extension Agent Capacity and Readiness to Adopt Policy, Systems and Environmental Change Approaches

**DOI:** 10.3389/fpubh.2022.856788

**Published:** 2022-05-26

**Authors:** Lisa Washburn, Heather Norman-Burgdolf, Natalie Jones, Lauren E. Kennedy, Soghra Jarvandi

**Affiliations:** ^1^Department of Family and Consumer Sciences, Institute of Agriculture, University of Tennessee, Knoxville, TN, United States; ^2^Department of Dietetics and Human Nutrition, University of Kentucky, Lexington, KY, United States; ^3^Family and Consumer Sciences Extension, University of Kentucky, Lexington, KY, United States; ^4^Health and Nutrition Institute, Michigan State University Extension, East Lansing, MI, United States

**Keywords:** PSE, Cooperative Extension, public health, PSE change, family and consumer sciences, Consolidated Framework for Implementation Research, health education, PSE barriers

## Abstract

**Introduction:**

Enhanced Extension outreach strategies combine traditional direct education programs with public health approaches like policy, systems, and environmental (PSE) change. However, the Cooperative Extension system and county-based Family and Consumer Sciences (FCS) Extension agents have historically prioritized direct education programming and diffusion of enhanced outreach strategies has varied. Extension personnel may lack capacity and readiness for successful PSE change implementation. This study explored perceived acceptability, capacity, and readiness for PSE change work among FCS Extension agents in two states.

**Method:**

A survey was developed framed by selected domains from the Consolidated Framework for Implementation Research: Intervention Characteristics, Inner Setting, Characteristics of Individuals, and Process. All questions utilized a 5-point Likert scale, except for an item examining respondents' stage of change regarding PSE change strategies. Descriptive statistics and response frequencies for all variables were calculated.

**Results:**

Survey responses (*n* = 116) indicated PSE change work was perceived as valuable. Potential barriers included perceived complexity, organizational readiness issues (e.g., reporting and evaluation structures; performance incentives), and worries about stakeholder responses in shifting away from direct education. Responses indicated self-efficacy for skills important in implementing PSE change. Most respondents (53%) indicated being at the pre-contemplation or contemplation stage of change in pursuing PSE change work.

**Discussion:**

Combining PSE change strategies and direct education programming allows Extension to do what it does best – provide effective programs to improve and sustain health and wellbeing of individuals and families. Findings are informative for others aiming to build capacity within community educators, Extension and public health professionals to implement PSE change.

## Introduction

The Cooperative Extension System (Extension) is a nationwide network providing community-based, nonformal education through local Extension offices affiliated with states' land grant universities. Extension has sought to improve quality of life and wellbeing for individuals and communities for more than 100 years by extending research findings from the university to communities through a partnership between federal, state, and local governments (1). While Extension's mission has been characterized as “Taking the University to the People” (2), a top-down paradigm, local Extension efforts take a grassroots approach characterized by county-based Extension agents working in partnership with residents and other stakeholders to solve problems. With offices in or near most U.S. counties, the footprint of county Extension offices and personnel differs by state (3). The Extension model and nationwide infrastructure have been envied by the healthcare sector, inspiring transformational ideas to increase healthcare access both parallel to and in collaboration with local Extension offices (1, 4, 5).

Agriculture, youth development, community development, and family and consumer sciences have been the primary focus for Extension education efforts. Campus-based, subject-matter Extension specialists train and support county-level educators known in some states as Extension agents. Local Extension agents identify and address community needs through education and outreach typically delivered through direct education and demonstrations (6). However, in the last decade Extension has increasingly focused on spurring long-term, sustainable changes requiring diverse partnerships, coalitions, and enhanced collaboration.

Family and Consumer Sciences (FCS) programs focus on enhancing community resilience and improving quality of individual and family life. In most states, FCS Extension addresses nutrition and food safety, human development, financial management, and health content areas. FCS Extension agents are skilled users of direct education methods reflecting Extension's traditional, expert-model for program delivery (7). Direct education methods usually address the individual or interpersonal/family level of the social ecological model and aim to increase knowledge and change behaviors (8). An enhanced approach combines both direct education and public health approaches like policy, systems, and environmental (PSE) change strategies. PSE change strategies extend benefits beyond individuals and families attending educational programs to communities, producing long-term, sustainable community health improvements (9).

Direct education and PSE change are more effective when implemented together (10). Enhanced approaches leveraging FCS direct education and public health strategies enable impacts at multiple social ecological model levels, including social networks, living and working conditions, and political factors determining individual health behaviors and community health outcomes (11). For example, PSE change strategies can include price change (e.g., improving pricing of healthier food items over less healthy items), space redesign, altering social norms, community empowerment, and redistributing resources (12). Enhancing healthier lifestyle supports for all community members can help alleviate some structural and social determinants of health known to exacerbate health inequities (12–14).

Supplemental Nutrition Assistance Program Education (SNAP-Ed) and other federally funded initiatives, such as the Centers for Disease Control and Prevention (CDC) High Obesity Program (HOP), have largely driven the increased emphasis on PSE change strategies in Extension. SNAP-Ed-funded Extension programs were mandated to provide comprehensive nutrition education programs including PSE change interventions starting in 2010. Extension PSE change approaches implemented through SNAP-Ed have evolved to address social determinants of health by promoting policy, systems and environmental changes impacting places where people live, work, and play (13, 15). In 2014 through HOP, CDC began funding Extension work specifically to address PSEs in counties with adult obesity rates over 40% (16). Additional Extension efforts to integrate PSE change with direct education include the Well Connected Communities Initiative, a partnership between the Cooperative Extension System and National 4-H Council funded by Robert Wood Johnson Foundation. The initiative supports systems change to advance health equity and improve social determinants of health (17).

The shift to PSE change work from primarily delivering direct education has challenged some county-based Extension personnel and state-level specialists, particularly those with a longer tenure within the traditional Extension system (18). Although Extension has been engaged in PSE change work more than 10 years, exploration of perceived acceptability among Extension professionals charged with implementation has been limited primarily to SNAP-Ed and other special grant-funded projects. For example, capacity limits noted among Extension and non-Extension SNAP-Ed implementers in one state included limited knowledge, training, resources, and experience with PSE change approaches (19). Others reported multi-level barriers to PSE change implementation, including lack of readiness among SNAP-Ed partner sites and implementers, and prioritizing direct education over PSE change work (20). Extension professionals' beliefs about the role of Extension may also pose challenges to PSE implementation (21). Participants in one national survey included Extension FCS, 4-H, nutrition education, and community development professionals; responses indicated varying levels of willingness to apply PSE change approaches in youth development activities. Most thought PSE change work would be a “big shift” in their work (22). Aside from this youth development focused survey, effort to understand agent viewpoints broadly within an Extension system has been limited.

The increased public and private investment into Extension to influence health-promoting PSE change and advance health equity make understanding perceptions and attitudes of FCS Extension agents critical. To better understand attitudes, perceptions, and readiness to implement PSE change strategies, we conducted a survey of FCS Extension agents in two neighboring states with similar trajectories for introducing PSE change into Extension work, including CDC's High Obesity Program (HOP) funding and SNAP-Ed PSE change implementation. This study explored perceived acceptability of PSE change work among FCS Extension agents in two states and broadly examined potential barriers and facilitators to advancing Extension's PSE change work.

## Method

### Sample

FCS Extension agents in Kentucky and Tennessee were invited to participate in an online survey in Spring 2020. Kentucky and Tennessee are neighboring states with similar obesity-related disease burdens, each comprised of primarily rural counties with few large metropolitan areas; both have county-based FCS Extension agents. Existing email listservs and distribution lists for each state were used for recruitment. Inclusion criterion was currently serving as an FCS Extension agent in Kentucky or Tennessee. Selection of a position title other than FCS Extension agent terminated the survey. There were no exclusions based on gender, race, or ethnicity.

### Survey Measures

We developed our survey by using selected domains and constructs of the Consolidated Framework for Implementation Research (CFIR) (23). The CFIR was developed based on review and synthesis of published implementation theories and provides a list of constructs within general domains thought to influence implementation. The CFIR, however, does not specify interaction between constructs and allows researchers to choose those most relevant for the study setting. The CFIR was selected because it can be used to guide formative evaluations and is well-suited for measuring complex, multi-level influences on implementation in real-world settings.

The CFIR includes 37 constructs in five domains: intervention characteristics, outer setting, inner setting, characteristics of individuals involved, and the process of implementation. As advised by CFIR developers, the research team reviewed CFIR domains and constructs, identified those of interest, and selected constructs most relevant for the study purpose and context (23) (see Table 1).

**Table 1:** Selected domains and constructs - consolidated framework for implementation research (23).

**CFIR Domain**	**Selected Constructs**
Intervention Characteristics *Characteristics of the intervention being implemented into a particular organization*	• Intervention source • Relative advantage • Adaptability • Trialability • Complexity
Outer Setting *The economic, political, and social context within which an organization resides*	• Patient (clientele) needs & resources • Peer pressure • External policies and incentives
Inner Setting *Structural, political, cultural, and organizational contexts through which the implementation process will proceed*	• Structural characteristics • Networks and communications • Culture • Implementation Climate - Tension for change - Learning climate • Readiness for Implementation - Available resources
Characteristics of Individuals *Characteristics of individuals involved with the intervention and/or implementation process, including cultural, organizational, professional, and individual mindsets, norms, interests, and affiliations*	• Access to information and knowledge • Knowledge and beliefs • Self-efficacy • Individual stage of change • Individual identification with organization
Process of Implementation *Interrelated series of sub-processes that may not occur sequentially*	• Planning • Executing • Reflecting and evaluating • Engaging

Research team members independently drafted questions in a shared document to address selected domains and related constructs, then jointly reviewed questions to clarify wording. After revision, the team reviewed the questions again by domain and construct to determine those most relevant and suitable given the survey purpose and sample. After this review, wording of survey questions was further refined. Questions and aligning domains/constructs were reviewed by a panel of FCS Extension agents and Extension specialists with PSE change experience and experts from the public health sector with knowledge of PSE change implementation and evaluation. All feedback was combined, and the survey was further refined by the research team based on suggested edits and reviewer comments. Questions utilized a 5-point Likert scale with response options from “strongly disagree” to “strongly agree” with a neutral midpoint (“neither agree nor disagree”) except for one item examining respondents' stage of change for PSE change implementation.

### Data Collection

Kentucky utilized the Qualtrics platform (Qualtrics.com); Tennessee used QuestionPro (questionpro.com). Both systems included an embedded document describing PSE change within the Extension context for review prior to completing the survey. Survey items were the same across both states. The survey invitation included a brief description of the survey, an attachment defining PSE change strategies within Extension contexts, and an embedded survey link. A reminder email was sent to non-respondents 1 week after the initial invitation. A subsequent final reminder email was sent 1 week later. FCS Extension agents were encouraged to participate regardless of exposure to or experience with PSE change work. Completion of the survey was considered consent to participate. Responses were anonymous; no incentives were offered. The Institutional Review Boards at the University of Kentucky and the University of Tennessee approved the study protocol.

### Data Analysis

Data from each state were cleaned and combined for analysis. Strongly agree and agree responses were collapsed to form a single category, as were strongly disagree and disagree responses (24, 25), resulting in three response categories: agree, neutral, and disagree. Variables were collapsed due to scarcity of data within the strongly agree and strongly disagree categories. Descriptive statistics and response frequencies for all variables were calculated. Surveys were analyzed using Microsoft Excel (version Microsoft 365).

## Results

### Participant Characteristics

A total of 116 FCS Extension agents completed the survey between Kentucky (*n* = 43) and Tennessee (*n* = 73). The sample was predominantly White, with a Master's degree or higher, mirroring the overall makeup of FCS Extension agents in both states. The largest percentage of participants had an educational background in Family and Consumer Sciences studies. Age and tenure with Extension was evenly distributed across both states (Table 2). The response rate for Tennessee and Kentucky, respectively, was 67.5 and 45.7%.

**Table 2:** Participant demographic characteristics (*n* = 116).

**Characteristics**	**Kentucky**	**Tennessee**	**Total**
	**(*n* = 43)**	**(*n* = 73)**	**(*N* = 116)**
**Age**, ***n*** **(%)**			
20–39	14 (33%)	35 (48%)	49 (42%)
40–59	21 (49%)	29 (40%)	50 (43%)
60–69	8 (19%)	9 (12%)	17 (15%)
**Race**			
Black /African American	1 (2%)	5 (7%)	6 (5%)
White	41 (95%)	64 (90%)	105 (92%)
Two or more mixed race/other	1 (2%)	2 (3%)	3 (3%)
**Education**			
Bachelor's degree	5 (12%)	24 (33%)	29 (25%)
Master's Degree or higher	38 (88%)	49 (67%)	87 (75%)
**Current role**			
County Director and / or Agent (FCS – 100% adult)	40 (93%)	51 (70%)	91 (78%)
County Director and / or Agent (FCS/4-H – split assignment)	1 (2%)	18 (25%)	19 (16%)
Other	2 (5%)	4 (5%)	6 (5%)
**Educational background**			
Child and family studies	10 (23%)	19 (26%)	29 (25%)
Food and nutrition	14 (33%)	21 (29%)	35 (30%)
Family and consumer sciences	19 (44%)	31 (42%)	50 (43%)
Health	1 (2%)	8 (11%)	9 (8%)
Other	-	3 (4%)	3 (3%)
**Extension employment, years**			
<5 years	5 (12%)	27 (37%)	32 (28%)
5–15 years	18 (42%)	18 (25%)	36 (31%)
15–25 years	10 (23%)	14 (19%)	24 (21%)
≥25 years	10 (23%)	14 (19%)	24 (21%)
**County of work**			
Rural	36 (84%)	59 (81%)	95 (82%)
Urban	7 (16%)	14 (19%)	21 (18%)

### Intervention Characteristics

**Figure 1:**
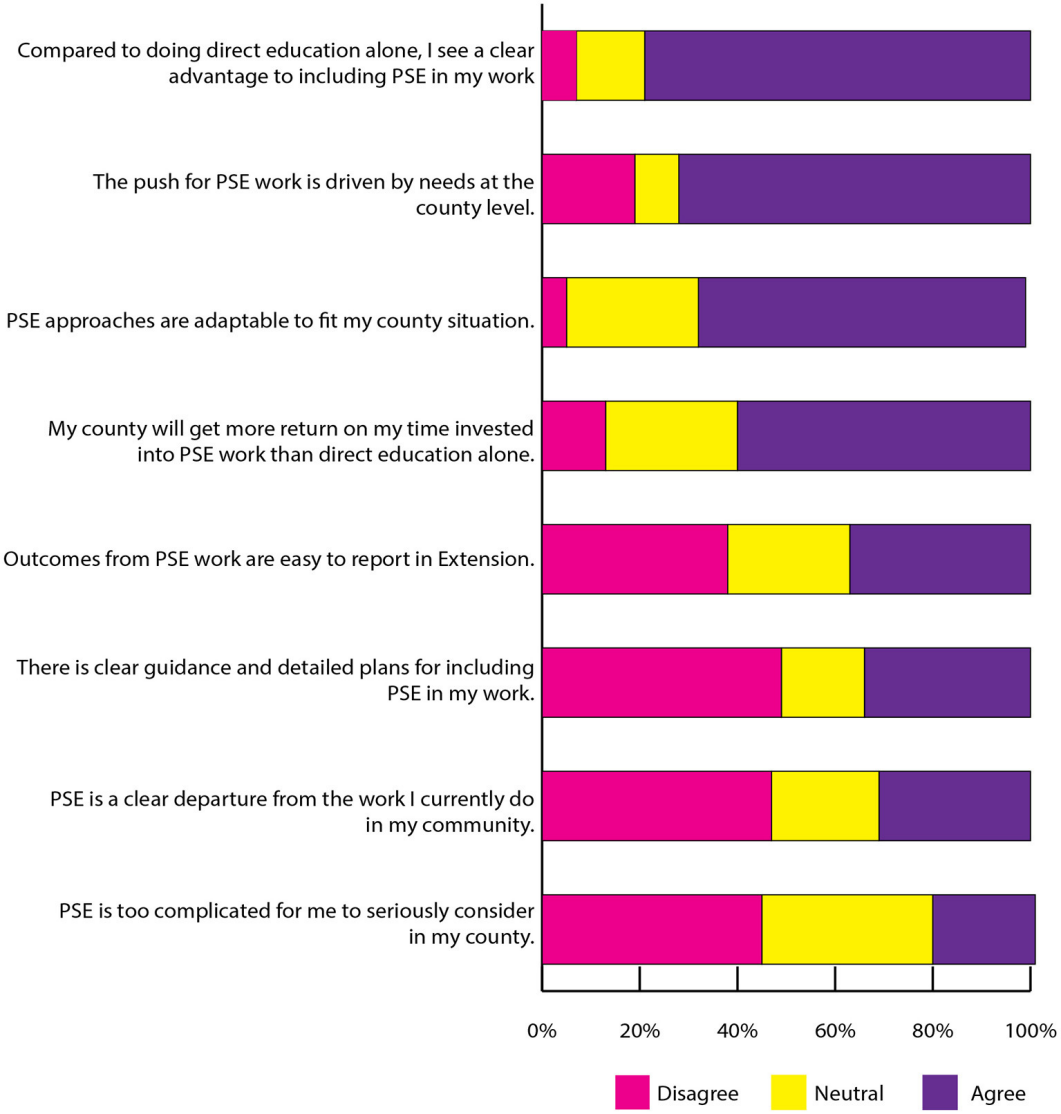
Intervention characteristics.

Overall, responses indicated FCS Extension agents perceived PSE change work as valuable. Analyses showed high levels of agreement for survey items related to perceived value of PSE change for community health (83%) and intervention source, adaptability, and relative advantage (see Figure 1, Intervention Characteristics). Lack of clear guidance and plans, and difficulty reporting outcomes, contributed to perceptions of perceived complexity of PSE change work, a potential barrier. Fewer agreed (21%) PSE change strategies are too complicated to seriously consider for their counties, but more than one-third were neutral.

### Inner Setting

**Figure 2:**
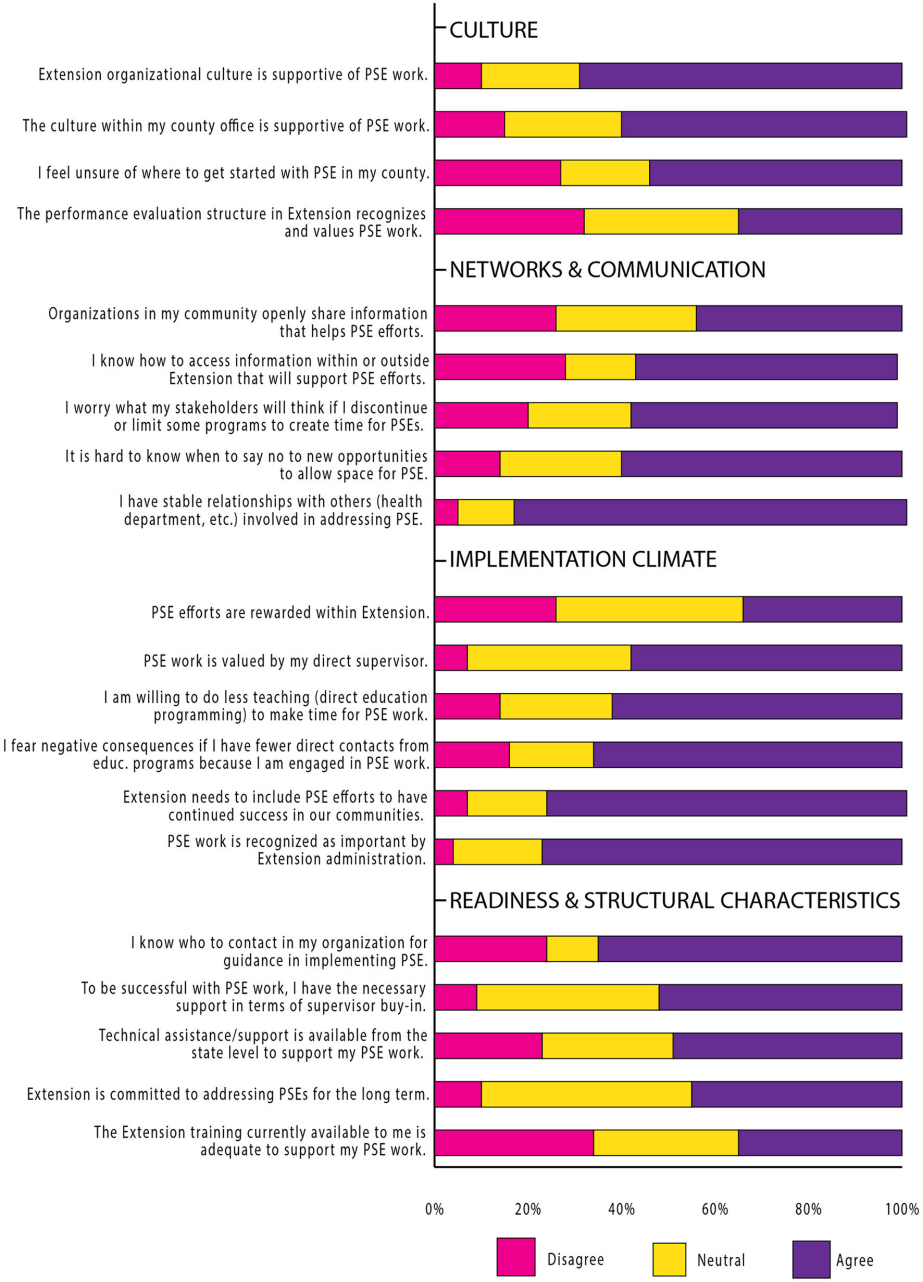
Inner setting.

Survey items addressing the CFIR domain inner setting had lower agreement and higher neutrality (response of “neither agree nor disagree”) than other domains, particularly for readiness and structural characteristics (see Figure 2, Inner Setting). For example, 45% of respondents agreed Extension is committed to addressing PSE changes for the long term, while 45% were neutral.

Implementation climate, a construct of the inner setting domain, generated incongruent responses. Respondents largely agreed Extension needs to include PSE change efforts for local success (77%) and Extension administration recognizes PSE change work as important (77%). However, only 34% agreed PSE change efforts are rewarded within Extension; 40% were neutral. Responses for learning climate items, an implementation climate sub-construct, indicated 62% of respondents are willing to shift from direct education to allow for PSE change work, but 66% have fears about negative consequences for changes in program outputs (direct contacts) likely resulting from such a shift. Similarly, responses for networks and communication items, an inner setting construct, indicated a majority (60%) have difficulty determining how to prioritize PSE change opportunities over other activities; 57% worried about stakeholder responses if some programs are limited or discontinued to allow for PSE change work (see Figure 2, Inner Setting).

A majority (69%) agreed the Extension organizational culture was supportive of PSE change work; fewer (61%) agreed the respondent's county office culture was supportive. Responses were nearly evenly divided regarding recognition and value of PSE change work in the Extension performance evaluation structure: 35% agreed PSE change work was valued and recognized, 32% disagreed, and 33% were neutral (see Figure 2).

### Characteristics of Individuals

**Figure 3:**
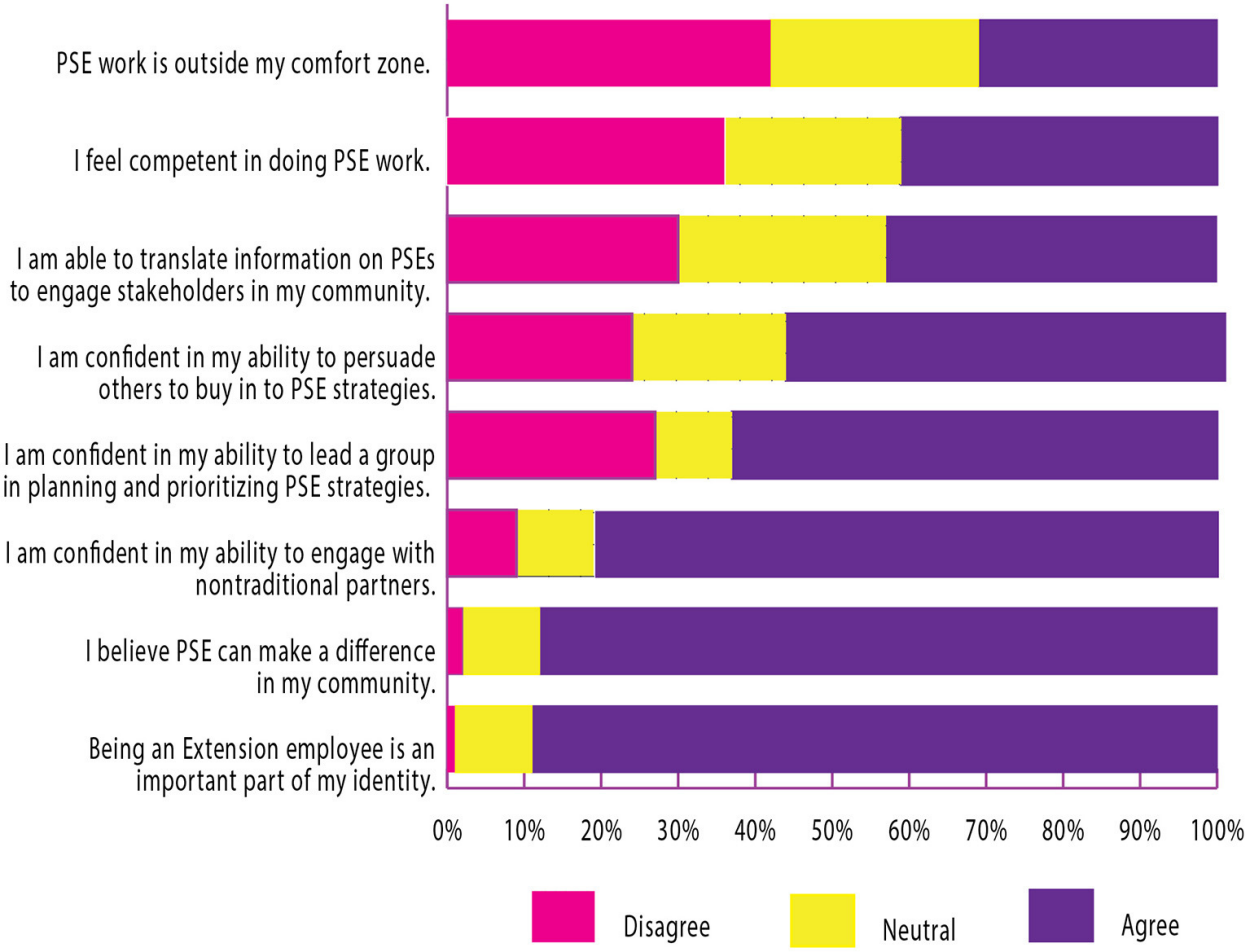
Characteristics of individuals.

Overall, most respondents have self-efficacy for skills important in implementing PSE change work: engaging with non-traditional partners (81%), leading a group in planning and prioritizing strategies (63%), and persuading others to buy in (57%) (see Figure 3). A majority (88%) agreed PSE change can make a difference in their communities, but lower agreement existed for other survey items addressing the knowledge and beliefs construct of the characteristics of individuals domain. For example, only 41% of respondents felt competent in doing PSE change work; 31% said PSE change is outside their comfort zone. Responses to these questions also had a higher neutrality compared to others in the same domain. Most respondents (53%) indicated being at the pre-contemplation or contemplation stage of change in pursuing PSE change work in their counties; few indicated action (22%) or maintenance (9%) stages.

### Process

Survey responses for process domain items were conflicting. Despite a minority (18%) indicating awareness of how PSE change efforts are evaluated, 58% said they can effectively share PSE-related successes in their communities. Only 27% agreed with the statement, “I see myself as a champion for PSE change in my community.” Nearly half (48%) indicated neutrality.

## Discussion

Extension has historically worked at the community level to speed adoption of innovations, like PSE changes, by working through Extension agents and with local coalitions and partners. The Extension System and has more than a decade of experience diffusing PSE change strategies (10, 26). The CDC-funded HOP is limited to a small number of qualifying counties, yet successfully implemented PSE change strategies from HOP have diffused across counties. Until now, FCS Extension agent capacity to conduct PSE change work was largely assumed without investigating acceptability and readiness to include PSE change approaches in county programming. Study findings provide insights into FCS Extension agent readiness to implement PSE change work and highlights barriers to adoption of PSE change strategies identified by respondents.

Overall, participants perceived organizational (Extension) support for PSE change approaches but also conveyed doubts about Extension's long-term commitment. This finding is consistent with results reported in a national survey of Extension professionals – 38% felt PSE change was a fad, potentially reflecting broader sentiments about Extension's involvement in community change work (22). Uncertainty regarding Extension's long-term commitment to PSE change work could be driven by continued reliance on grant funding (e.g., SNAP-Ed and CDC) and slow pace of organizational change, particularly within reporting and evaluation structures valuing direct education over sustained change through implementation of PSE change strategies (20).

### Extension Agent Readiness

Extension agents are instrumental facilitators of county-level PSE change work (27–29) but come to Extension with varying academic backgrounds and experiences typically excluding public health (30). FCS Extension agents traditionally prioritize direct education programming addressing nutrition, health, and wellbeing. Similar prioritization of direct education has been noted among SNAP-Ed implementers specifically tasked with supporting PSE strategies (20). Skills for effective educational program delivery differ from the skills, support, and resources required for successful PSE change strategy implementation. PSE change work is valued within the national Extension system (29, 31) but implementation varies between counties and states likely due to knowledge and skill gaps among county-based Extension agents.

Agreement for relative advantage, relative priority, and adaptability suggest FCS Extension agents believe PSE change work is important for community change and valuable for Extension (32). However, high neutrality in several CFIR domains may indicate gaps in basic understanding of PSE change noted in other surveys of Extension professionals (33). PSE change work is inherently more complex than direct education methods. Training needs in PSE change terminology, concepts, implementation, and evaluation have been noted for SNAP-Ed and EFNEP professionals and others implementing PSE change (20, 33–35). In this study, 35% neutrality on the question, “PSE change work is too complicated for me to seriously consider in my county” suggests some had not pondered PSE change implementation. Neutral and agree responses combined (56%) nearly match the 53% indicating being at pre-contemplation or contemplation stages in pursing PSE change work in their counties.

Findings regarding PSE change evaluation were incongruent. A majority (58%) agreed they can effectively share successes related to PSE change in their communities, but only 37% agreed PSE change outcomes are easy to report. Notably, fewer than one in five participants were aware of how PSE change efforts are evaluated. Other surveys of Extension professionals involved in PSE change work revealed uncertainty about what qualifies as a PSE change strategy and inability to identify PSE change efforts in their communities (33). Gaps in knowledge of evaluation methods, reporting, and sharing successes suggest additional training and resource needs for evaluation, a finding consistent with other studies (36).

### Organizational Barriers

Despite high levels of agreement that the Extension organizational culture is supportive of PSE change work, survey findings suggest notable internal barriers limiting adoption. These barriers illuminate opportunities to support county-level PSE change adoption, like streamlining outcome reporting and increasing training, support, and technical assistance for implementation and evaluation.

Complexity and perceived relative priority within the organization are barriers to PSE change implementation (32). Worries about negative consequences from fewer direct contacts and lower levels of agreement regarding internal recognition for PSE change work may reflect perceptions that direct education is valued over PSE change the organizational level. Most participants had difficulty declining direct education opportunities to allow space for PSE change work. In many states, including those in this study, Extension agents have high levels of autonomy in choosing programmatic focus in their communities. Local demand for Extension programs typically exceeds capacity. The array of programmatic options available to Extension agents may be overwhelming (37). These contextual factors may contribute to FCS Extension agents' challenges in prioritizing PSE change work.

Additional barriers included worries about stakeholder perceptions with modifications to plans of work, fewer direct education activities to increase PSE change efforts, and performance evaluation structures not recognizing or valuing PSE change work. Despite system-level efforts to value outcomes over number of people reached, direct contacts remain a metric of success. Fears of negative consequences from program changes resulting in fewer direct contacts may limit adoption. Inaccurately perceived negative consequences for reduced contacts resulting from PSE change work, compared to direct education, can be corrected through clear communication from upper administration and those performing personnel evaluations (38).

Expectations for implementing multi-level approaches should be included in job descriptions and clarified in scope of responsibility for current employees (39). Two questions in the characteristics of individuals domain regarding identification with the organization (“Being an Extension employee is an important part of my identity”) and knowledge and beliefs (“PSE change can make a difference in my community”) garnered the highest levels of agreement on the survey. Because direct education is traditionally preferred and prioritized, Extension professionals may also prize their teaching role and claim this as part of their identify, causing potential conflict with expectations for PSE change work despite recognizing value in these approaches. Cognitive dissonance between the direct educator and PSE implementer role may be remedied by presenting a “program plus PSE” approach, where traditional educational programs are enhanced with PSE change interventions. Emphasizing advantages of PSE change over direct education alone while also acknowledging complexity may help personnel feel more confident and enhance compatibility of PSE change alongside the educator role (18, 32). Support for collaborative, multi-level approaches is essential to motivate personnel to tackle complex community-level issues.

### Organizational Readiness

SNAP-Ed and EFNEP professionals have been the focus of several published studies about readiness for PSE change within Extension, an understandable focus given federal mandates to incorporate PSE change with direct education (19, 34, 40, 41). An unintended consequence, however, may be views among Extension agents erroneously limiting scope of PSE change efforts to SNAP-Ed and EFNEP eligible sites and participants. Opportunities to engage in PSE change work exist across all program areas within Extension. The need for non-SNAP-Ed partners has been documented (40). Adequately addressing social determinants of health and health equity within communities requires new approaches to translate and demonstrate PSE change in settings unaffiliated with nutrition education programs. Similarly, strategies aimed beyond increased access to healthy foods and physical activity, the primary focus thus far, must be demonstrated (14).

In the past, PSE change work absent an intentional focus on equity perpetuated health disparities in communities (often predominantly Black, Indigenous, and People of Color) made vulnerable by historical, ongoing economic disinvestment, social exclusion, and systemic oppression. Deliberate action by Extension personnel to address structural and social determinants of health, like racism and power, must be included in any PSE change work. In 2021, Extension published a new National Framework for Health Equity and Wellbeing (Framework) identifying and outlining health equity, social determinants of health, and community engagement as core themes in a national approach to health programming (31). The new Framework offers an opportunity to support state and local personnel in aligning PSE change work with established metrics of health equity (42–44).

In Extension, PSE change is often touted as a valuable approach to changing contexts that shape health outcomes, rather than affecting individual behavior through education alone. However, Extension is not immediately equipped to address PSE change failures leading to health inequities (45–47). The relative racial, ethnic, and gender homogeneity of Extension personnel represented in this study sample mirror personnel found at most 1862-designated land grant universities. Personal and individual biases, coupled with organizational and institutional racism and power differentials, require significant investment in organizational and individual capacity building for Extension staff at all levels if PSE change work is to address health equity and not cause further harms. As capacity or readiness improves, frameworks, such as R4P, Bounded Justice, or Collective Healing, can guide community-led PSE change processes toward health equity (45, 48, 49).

These findings are informative for entities working with Extension to implement PSE change strategies and highlight important considerations for rolling out large initiatives and working with diverse communities. Extension personnel have varying degrees of readiness to engage in PSE change work shaped by organizational and community factors, personal attitudes and biases, and educational/professional backgrounds. An organizational pivot to prioritize PSE change and a significant influx of resources (funding, training/education, partnerships) is needed for broad implementation within the national Extension system. Partner organization personnel should critically examine assumptions about individual and organizational readiness and seek to understand attitudes and perceived barriers of Extension personnel within community contexts.

### Limitations

This study has several limitations. First, the survey has not been validated for assessing beliefs and perceptions regarding PSE change work; however, using a validated framework and constructs for survey development was intentional for reducing bias. Second, our sample size was relatively small and the response rate may have been impacted by the onset of the COVID-19 pandemic during spring 2020. Thus, we cannot generalize findings and are unable to assess the potential differences among FCS Extension agents, for example, by years of experience. Third, data were collected from FCS Extension agents irrespective of previous knowledge or experience about PSE change. While this was intentional to fully understand baseline knowledge and perceptions, the high degree of neutrality in responses may result from questions not being applicable to all respondents. Because survey content related to job duties, respondents may have perceived some responses to be more acceptable than others, introducing social desirability bias.

## Conclusion

PSE change implementation may appear daunting as a new endeavor for FCS Extension agents. However, the outcomes of equitably implemented PSE change strategies are beneficial for health promotion and are worth pursuing. Combining PSE change work with traditional Extension direct education programming allows Extension to do what it does best—provide research-based, effective programs and interventions to improve and sustain the health and wellbeing of individuals and families. Integrating PSE change within Extension aligns our work with the growing body of evidence showing PSE changes lead to sustained positive health outcomes, providing a novel mechanism for Extension to partner with public health entities for improved community health. Our findings provide unique insight informative for other Extension and public health entities looking to build capacity within community-level educators, Extension personnel, and public health professionals to implement PSE change work. Moving forward, Extension should ensure PSE change strategies are presented as a priority to all staff within the organization.

## Data Availability Statement

The raw data supporting the conclusions of this article will be made available by the authors, without undue reservation.

## Ethics Statement

This study was reviewed and approved by University of Tennessee Institutional Review Board and University of Kentucky Institutional Review Board. Written informed consent for participation was not required for this study in accordance with the national legislation and the institutional requirements.

## Author Contributions

LW, HN-B, NJ, and LK jointly developed survey instrument and study design. LW, HN-B, and NJ coordinated data collection and contributed to data interpretation. SJ conducted data analysis. LW led manuscript development. All authors contributed to read and approved the final manuscript.

## Conflict of Interest

The authors declare that the research was conducted in the absence of any commercial or financial relationships that could be construed as a potential conflict of interest.

## Publisher's Note

All claims expressed in this article are solely those of the authors and do not necessarily represent those of their affiliated organizations, or those of the publisher, the editors and the reviewers. Any product that may be evaluated in this article, or claim that may be made by its manufacturer, is not guaranteed or endorsed by the publisher.
